# Clinicopathologic Features, Surgical Treatment, and Pathological Characterization of Canine Dacryops with Different Localization

**DOI:** 10.3390/vetsci12080705

**Published:** 2025-07-28

**Authors:** Barbara Lamagna, Luigi Navas, Francesco Prisco, Dario Costanza, Valeria Russo, Francesco Lamagna, Cristina Di Palma, Valeria Uccello, Giuseppina Mennonna, Orlando Paciello, Flaviana La Peruta, Giovanni Flauto, Giovanni Della Valle

**Affiliations:** 1 Unit of Surgery, Department of Veterinary Medicine and Animal Productions, University of Naples “Federico II”, 80137 Naples, Italy; blamagna@unina.it (B.L.); lnavas@unina.it (L.N.); francesco.lamagna@unina.it (F.L.); cristina.dipalma@unina.it (C.D.P.); dr.flavianalaperuta@gmail.com (F.L.P.); 2 Unit of Pathology, Department of Veterinary Medicine and Animal Productions, University of Naples “Federico II”, 80137 Naples, Italy; francesco.prisco@uzh.ch (F.P.); valeria.russo@unina.it (V.R.); paciello@unina.it (O.P.); 3 Interdepartmental Center of Veterinary Radiology, University of Napoli “Federico II”, 80137 Naples, Italy; dario.costanza@unina.it; 4 Veterinary Center ASL Napoli 1, 80145 Naples, Italy; dot.uccellovaleria@gmail.com (V.U.); pina.mennonna@gmail.com (G.M.); 5 ASL Napoli 2 Nord, 80027 Naples, Italy; flautovet@gmail.com

**Keywords:** canine, computed tomography, dacryops, dacryocystography, dogs, immunohistochemistry, third eyelid

## Abstract

Lacrimal cysts (dacryops), which involve lacrimal tissue, are uncommon in dogs with an obscure/unclear pathogenesis. Compared to the current available literature, this report describes the clinicopathologic and immunohistochemical features of two cases of unusual dacryops in brachycephalic dogs. A three-year-old male Cane Corso was referred with a 1-month history of swelling ventromedial to the left eye associated with blepharospasm and epiphora. Furthermore, a severe lower and upper eyelid entropion and a deep corneal ulcer were present. B-mode ultrasonography and a CT scan revealed a subcutaneous cyst, closely adherent to the maxillary bone. Surgical removal and the correction of entropion were performed. No recurrence and/or complication was detected by seven-year follow-up. Histopathology revealed a cystic structure with single- to double-cell-layered, nonciliated, cuboidal epithelia. Alcian blue stain revealed rare, disseminated goblet cells admixed with epithelial cells. The epithelium was strongly Cytokeratin-positive by immunohistochemistry and appeared lined by several layers of smooth muscle actin (SMA)-positive myoepithelial cells. A 1-year-old male French Bulldog with a 3-month lesion of the third eyelid of the right eye. The lesion (15 mm × 7 mm) beneath the conjunctiva appeared pale-pink, smooth, and multilobulated. Excision was performed by blunt dissection through the conjunctiva on the palpebral surface of the third eyelid. Recovery was uncomplicated, and no recurrence has been noted at three-year follow-up. Cytology of the cystic fluid and histopathology and immunohistochemistry of the cyst wall revealed findings for case 1. To further characterize the SMA-positive spindle cells located directly beneath the cyst-lining epithelium, double-color immunofluorescence for SMA and p63 (a myoepithelial cell marker) was performed on the sample from case 2. The analysis revealed that the SMA-positive cells lacked p63 expression, indicating a non-myoepithelial phenotype. The histological findings in our cases are consistent with previous reports of canine dacryops. The positivity of immunohistochemical staining for SMA in cells directly beneath the epithelium of dacryops in the cases here described in two brachycephalic dogs is consistent with previous reports in dogs and horses but in contrast with a retrospective study about a human dacryops. These results support the conclusion that the pathogenesis of dacryops in dogs should exclude failure of ductular “neuromuscular” contractility.

## Simple Summary

This study looked at a rare problem in dogs called dacryops, which are fluid-filled cysts that form in the lacrimal tissue. The exact cause of these cysts is still not fully understood. The researchers described two unusual cases in the brachycephalic dog. One case involved a Cane Corso with swelling, pain, and a corneal ulcer caused by the cyst. The second case was a French Bulldog with a lump on the third eyelid. In both cases, the cysts were successfully removed through surgery, and the dogs recovered without any complications or recurrence after more than two years. Histopathology showed different layers of SMA-positive spindle cells directly beneath the epithelium lining the cyst. These findings were similar to what has been seen in dogs and horses but different from what has been found in humans. The study suggests that in dogs, these cysts are probably not caused by failure of ductular neuromuscular contractility. Understanding the nature of these cysts and how to treat them can help veterinarians manage similar cases better in the future, especially in breeds that are more likely to develop such conditions.

## 1. Introduction

Dacryops is a cystic ectasia of the lacrimal glandular duct (both intraglandular and extraglandular). Canaliculops, or canaliculocele, is a term used for a distention of a lacrimal canaliculus. Dacryops and canaliculops are rare in dogs. To date, only 19 cases of dacryops [1,2,3,4,5,6,7,8,9,10,11] and three cases of canaliculops [1,12,13] have been described in the literature in this species.

Furthermore, such diseases are exceptionally uncommon in other domestic species, and only three reports have been published in cats: one orbito-nasal cyst [14], one dacryops of the third eyelid lacrimal gland [15], and one case of bilateral temporal dacryops [16]. One report of a dacryops localized close to the nasolacrimal duct in a 16-year-old horse [17] has been described. Interestingly, an orbital lacrimal cyst in a 23-year-old female red-eared slider (Chrysemys scripta elegans) was reported [18].

In human medicine, a 2002 review of the literature revealed only 6 reported cases of simple dacryops in a decade and described a case series of three patients affected [19]. Canaliculops, or canalicocele, also appears as an extremely rare disorder, with only 6 reports substantiated by histopathological examination in the human medicine literature [20]. Instead, only one case of congenital canalicular cyst (lined mostly by canalicular epithelium, expressing a cytokeratin profile similar to that of a normal canaliculus and canaliculops) attached to a small bony spicule with no connection to the lacrimal sac or canaliculus was described in a 49-year-old man [21,22].

Pathogenetic theories of dacryops include dysfunction of the rich nerve plexus around the ductules, periductular inflammation leading to scarring, or a failure of ductular “neuromuscular” contractility [23]. However, in all species, the exact pathogenesis of dacryops remains obscure due to the fact that the structural characteristics of the cells constituting the linings of the lacrimal cysts have not been definitively clarified [23].

The first pathogenetic theory of dacryops, according to which inflammation or trauma blocked the excretory ducts, causing mechanical dilation, was experimentally refuted, as obliteration of the lumens caused atrophy of the gland and cessation of secretion without lumen enlargement [24]. Furthermore, histopathological examination of five cases of human dacryops showed immunoreactivity to IgA on the luminal surface of the epithelial ductal cyst, leading the authors to hypothesize that IgA hypersecretion, with an osmotic effect, may contribute to cyst formation [24].

For a clearer understanding of this pathology in all the species affected by it, the in-depth studies of the clinical, diagnostic, and therapeutic aspects in clinical cases acquire fundamental importance. Furthermore, the evaluation of the structural aspects of the cells that delimit the cystic dilatations of the lacrimal tissue can contribute to determining the pathogenetic aspects of this condition, with translational significance.

To this aim, we report below two particular cases of lacrimal cysts in two dogs, in which the diagnostic and therapeutic insights provide useful indications for a better understanding of this pathology and, at the same time, for adequate management in the clinical practice of affected patients.

## 2. Case 1

**Figure 1:**
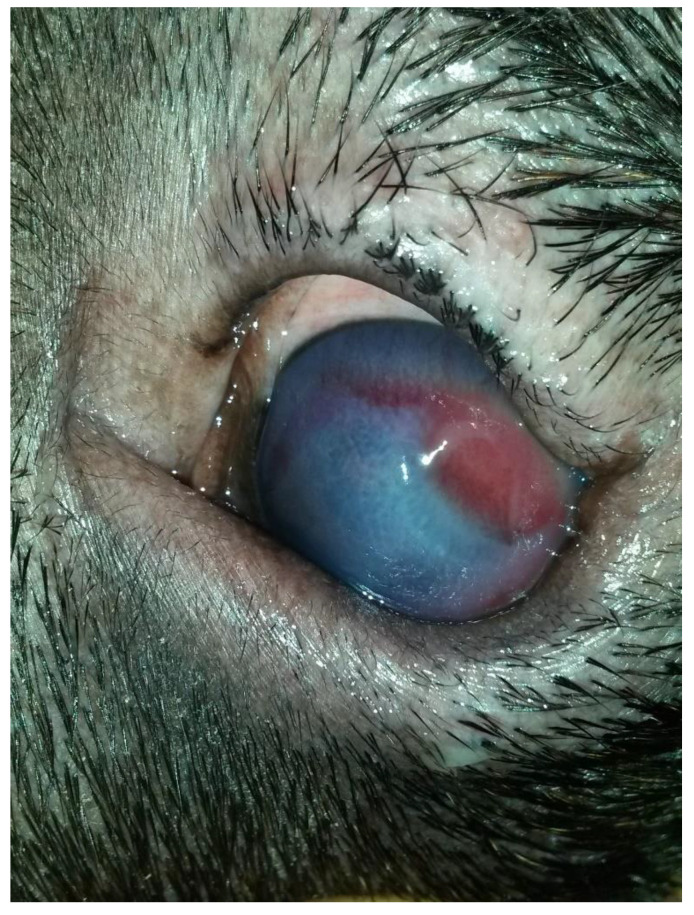
Case 1: A deep corneal ulcer is evident. Moderate corneal neovascularization, granulation tissue, and diffuse corneal edema are present.

**Figure 2:**
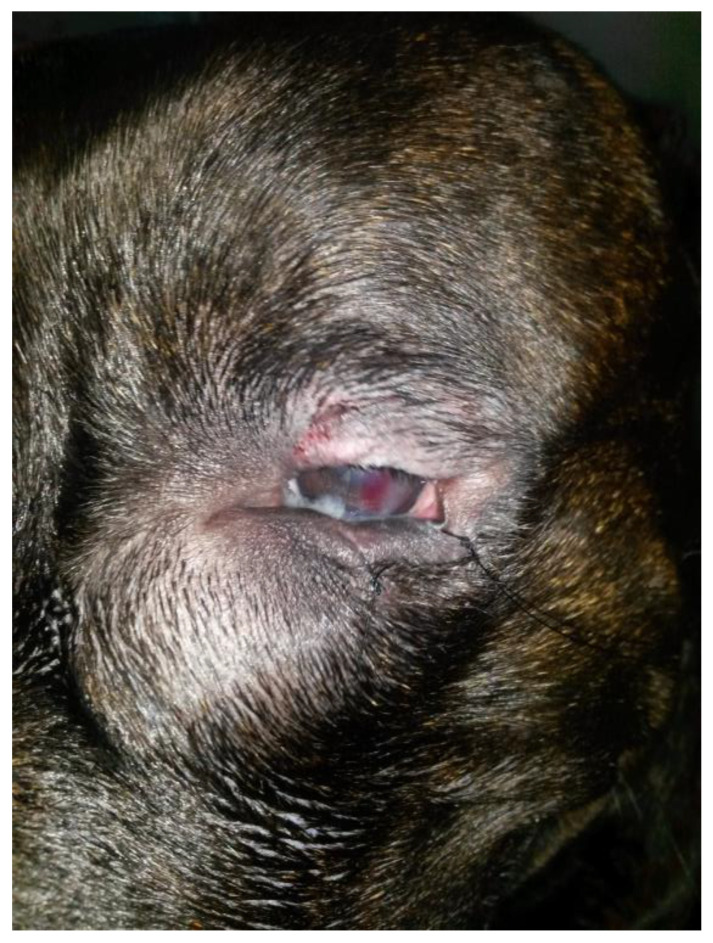
Case 1, after 7 days of topical treatment of the ulcer and tacking, showing the mass at the rostroventromedial aspect of the OS.

A three-year-old male Corso dog was referred with a 1-month history of swelling ventromedial to the left eye (OS) associated with blepharospasm and epiphora. One week before referral, a transparent fluid was aspirated, and the mass had disappeared. There was no record of any ocular or nonocular issues, trauma, or concomitant morbidities. At the time of referral, the dog was receiving tobramycin ophthalmic solution (Stilbiotic, Trebifarma s.r.l., Genova, Italy) every four hours, 5% acetylcysteine solution (Abinac, Trebifarma s.r.l., Genova, Italy) every two hours, and 1% atropine sulfate solution daily. A complete ophthalmic examination (OE) on both dog’s eyes was performed by an expert ophthalmologist (BL), including neuro-ophthalmological examination, STT-1 (Dina strip, DYASET s.r.l., Portomaggiore, FE, Italy), slit-lamp biomicroscopy (Kowa SL-15, Kowa Company Ltd., Nagoya, Japan), direct ophthalmoscopy (Panoptic ophthalmoscope, Welch Allyn), fluorescein staining (FioniaVet, Bjebto ApS, Odense, Denmark), and the Jones test. The OE revealed a severe lower and upper eyelid entropion and a deep corneal ulcer (that extended beyond 30% of the total corneal thickness) in the OS, which showed marked blepharospasm with severe conjunctival hyperemia and moderate epiphora (Figure 1 and Figure 2).

Schirmer tear test-1 (STT-1) results were 21 mm/min in the right eye (OD) and 30 mm/min in the OS. Intraocular pressure was not measured due to the dog’s temperament. The corneal lesion was a round, centro-temporal corneal ulcer that was approximately half of the stromal depth. Abundant corneal neovascularization, granulation tissue, and diffuse corneal edema were present. The Jones test was positive bilaterally. The swelling, described by the owners, was not evident at the referral examination.

The physical examination, complete blood count, and serum biochemical profile were normal.

A temporary eyelid tacking for entropion was performed on OS under sedation. The dog received an intramuscular (IM) administration of 0.02 mg/kg of acepromazine (Prequillan 10 mg/mL, Fatro Spa, Ozzano dell’Emilia, Italy) and 0.3 mg/kg of methadone (Semfortan 10 mg/mL, Dechra Pharmaceuticals, Skipton, UK). The procedure aimed to eliminate trichiasis, reduce corneal irritation, and relieve spastic entropion to plan surgical treatment for the following week. The therapy set at the referral time was followed for the other two weeks.

At the one-week follow-up, the swelling had recurred rostroventral medial to the OS. At the ophthalmic examination, in the OS, conjunctival hyperemia and corneal edema had significantly improved, the cornea was fluorescein negative, and the vascularization and granulation tissue were markedly reduced. In addition, there was a well-demarcated, ovoid, firm, non-ulcerated, subcutaneous mass measuring 25 mm × 15 mm adjacent to the medial canthus and medial inferior eyelid margin (Figure 2). The Jones test was positive in the OD and negative in the OS.

**Figure 3:**
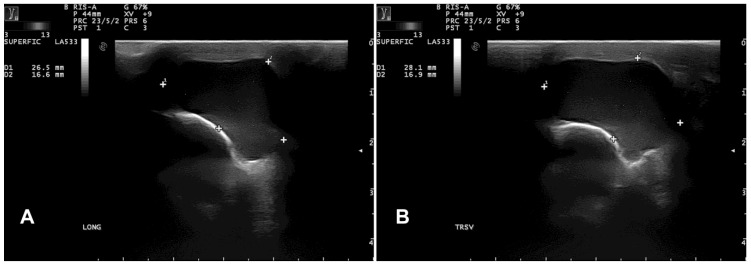
Case 1: longitudinal (**A**) and transversal (**B**) ultrasonographic images of a thin-walled cystic structure (enclosed by the dotted electronic callipers) containing finely echogenic, fluctuating material.

B-mode ultrasonography revealed a subcutaneous cyst characterized by a hypoechoic content lined by a thin wall (Figure 3).

**Figure 4:**
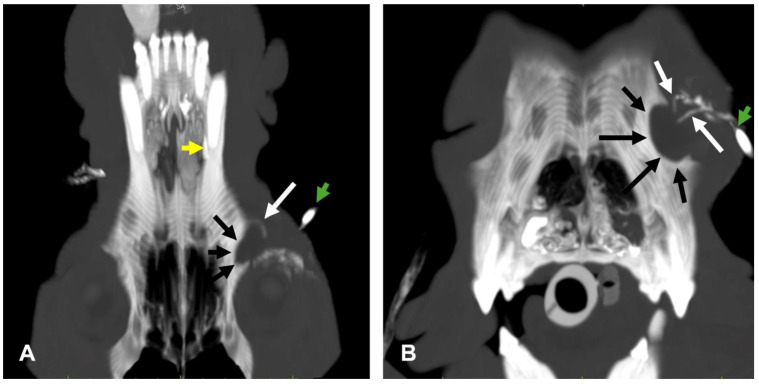
Case 1: left CT dacryocystography. (**A**) Dorsal 3D maximum intensity projection (MIP). The lacrimal duct (white arrow) is diverted by the cyst, and the periorbital portion of the left maxillary bone shows a compression lysis (black arrows). The contrast media injection site (green arrow) and the opening of the lacrimal duct within the ipsilateral nasal cavity ARE also visible (yellow arrow) (**B**) Axial 3D MIP. The superior and inferior lacrimal canaliculi of the lacrimal duct (white arrows) and the maxillary bone lysis (black arrows) are visible.

Left computed tomography dacryocystography delineated a normal nasolacrimal structure without any connection with the cystic lesion that was closely adherent to the maxillary bone (Figure 4).

The dog was premedicated with intramuscular (IM) administration of 0.02 mg/kg of acepromazine (Prequillan 10 mg/mL, Fatro Spa, Italy) and 0.3 mg/kg of methadone (Semfortan 10 mg/mL, Dechra Pharmaceuticals, UK). General anesthesia was induced using intravenous (IV) administration of propofol (2–4 mg/kg; Proposure 10 mg/mL, Merial, France) and, after endotracheal intubation, maintained with isoflurane (Isoflo^®^, Abbott, Chicago, IL, USA) in 100% oxygen and placed in sternal recumbency.

The periocular hairs were clipped. The periocular and ocular tissues were aseptically prepared with a 1:50 dilution of povidone-iodine (Viatris, Milan, Italy) solution.

A 25 mg/kg IV dose of Cephazolin (TEVA s.r.l., Milan, Italy) was administered before surgery. A sterile 24 g venous catheter (BD s.r.l., Milan, Italy) was used to cannulate the nasolacrimal duct.

**Figure 5:**
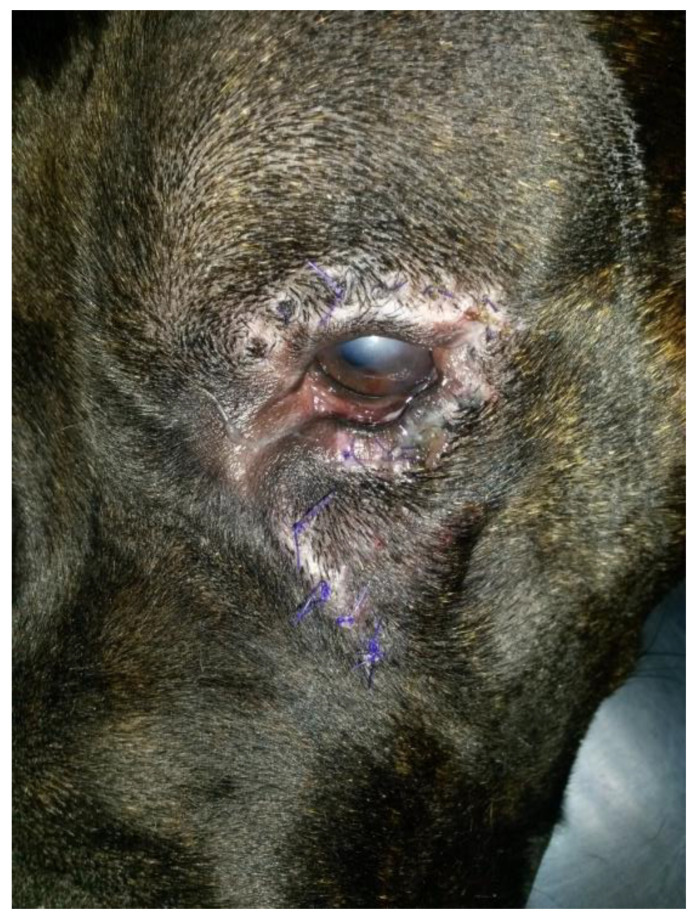
Case 1: 7 days after surgery (Hotz-Celsus modified entropion technique).

**Figure 6:**
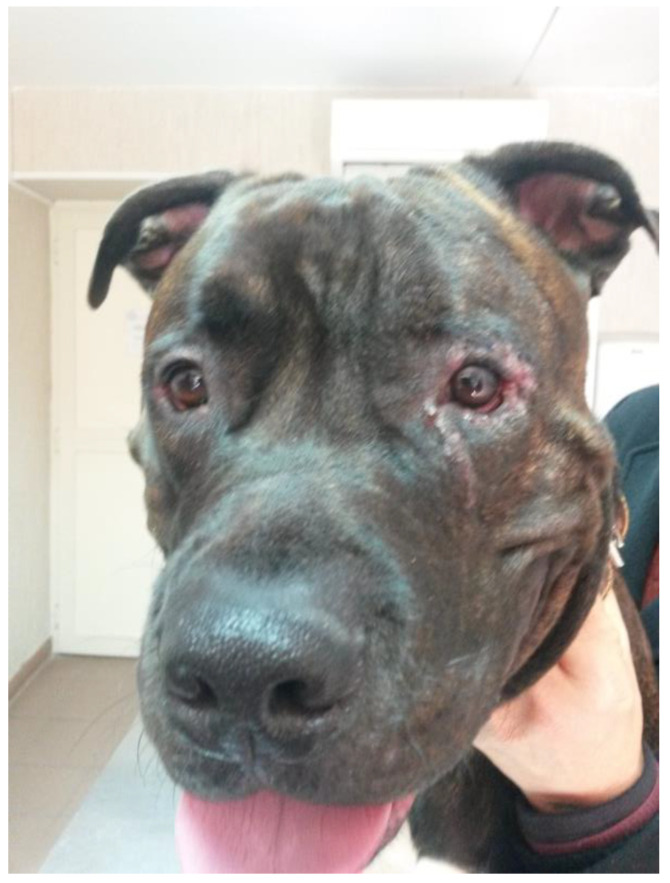
Case 1: 14 days after surgery.

A skin incision was made with a no. 11 scalpel blade over the cyst. The cyst was surgically removed, and the upper and lower entropion were corrected using the Hotz-Celsus modified entropion technique. During surgery, no punctate reflux of fluid was noted during manipulation of the cyst. No connection was evident between the cyst and the lacrimal sac or canaliculus. Although the cyst was carefully dissected free of its soft-tissue attachments, the rupture of the cystic wall occurred intraoperatively. Part of the viscous and transparent fluid was sampled. The subcutaneous tissues were closed using 4-0 polygalactin (Vicryl, Ethicon Inc., Raritan, NJ, USA) in a simple continuous pattern, and the skin was closed with 4-0 nylon (Nylon, Ethicon Inc., USA). The postoperative treatment consisted of carprofen (Rimadyl, Zoetis Italia s.r.l., Rome, Italy) at a dosage of 4 mg/kg of body weight on the day of surgery and SID for 4 days postoperatively, as well as amoxicillin and clavulanic acid (Synulox, Zoetis Italia s.r.l.) at a dosage of 10 mg/kg of body weight amoxicillin and 2.5 mg/kg of body weight clavulanic acid BID for 7 days postoperatively. Topical tobramycin (Stilbiotic, Trebifarma s.r.l., Genova, Italy) drops were applied in the conjunctival sac of the OS every six hours for a week. At the discharge, the Elizabethan collar was recommended until the reassessment. A one-week follow-up showed the surgical wound and cornea were healed; fluorescein was negative, and no complications were referred or detected. (Figure 5 and Figure 6). No recurrence was found at two weeks, one month, three months, 18 months, and at seven-year follow-up.

## 3. Case 2

**Figure 7:**
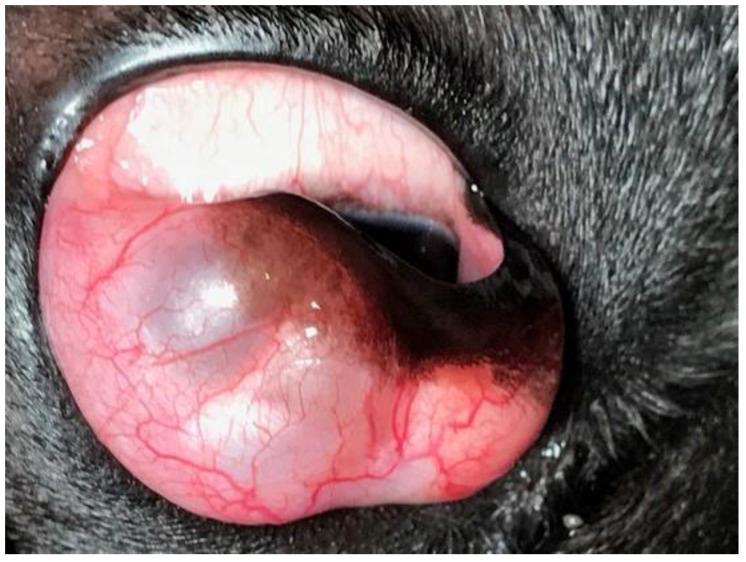
Case 2: French Bulldog, with a mass of 3 months duration on the third eyelid of OD.

A 1-year-old male French Bulldog, with a mass of 3 months duration on the third eyelid of the OD, was referred. The dog’s ophthalmic history did not include any ocular trauma or disease, nor surgery for replacement of the third eyelid prolapsed gland. The dog was receiving no topical or systemic medication. Ophthalmic examination, performed by BL as described for Case 1, revealed a mass located on the anterior aspect of the third eyelid of the OD, beneath the conjunctiva, which appeared pale-pink, smooth, and multilobulated (Figure 7).

Values of the STT-1 were 24 and 22 mm per minute OD and OS, respectively. The Jones test was positive bilaterally. Intraocular pressure was normal in both eyes (OD: 18 mmHg, OS: 20 mmHg). Physical examination, complete blood count, and serum biochemistry profile were normal.

**Figure 8:**
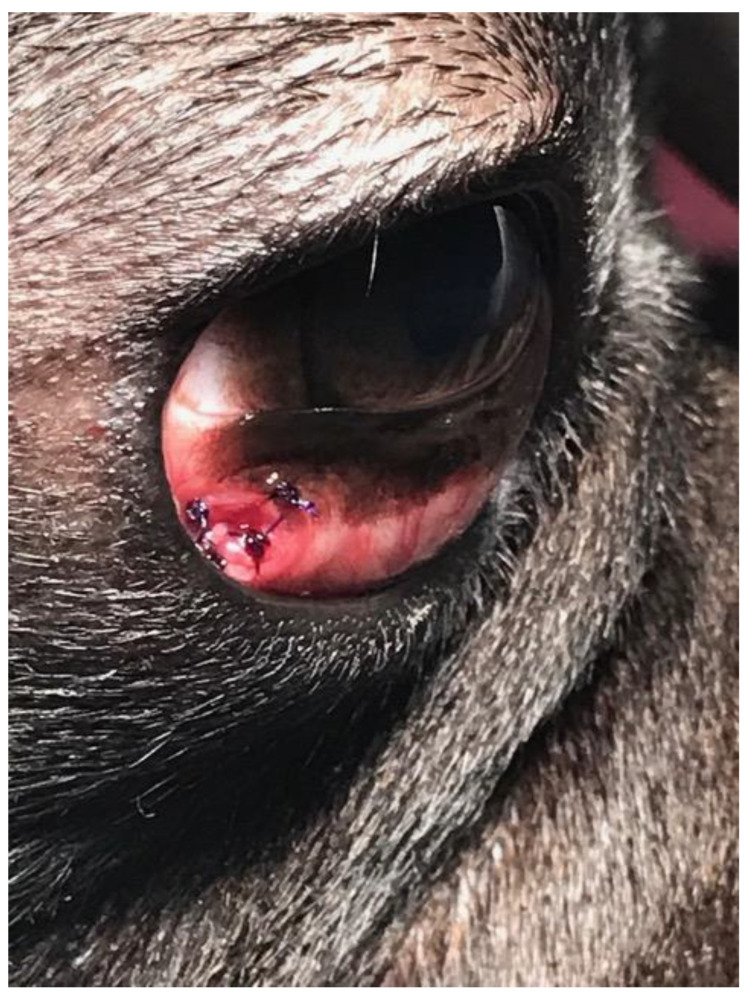
Case 2: Excision of the cyst was performed.

The dog was placed under general anesthesia, and the surgical field was prepared as described for case 1. After preparing the surgical site with a dilute povidone-iodine solution (1:50 dilution) and rinsing the eye with sterile saline, a blepharostat was placed to retract the eyelids. The third eyelid was fixed with Graefe forceps, so the palpebral conjunctival surface was excised on the cystic lesion. Cyst excision was performed by blunt dissection using tenotomy scissors through the conjunctiva on the palpebral surface of the third eyelid (Figure 8).

The cyst (15 mm × 7 mm), filled with clear fluid, was completely excised. Surgical wound closure was performed in an interrupted pattern with polydioxanone 6/0. Postoperative care was similar to that described for case no. 1. Recovery was uncomplicated, and no recurrence occurred at two weeks, one month, three months, and 12 months of follow-up.

**Figure 9:**
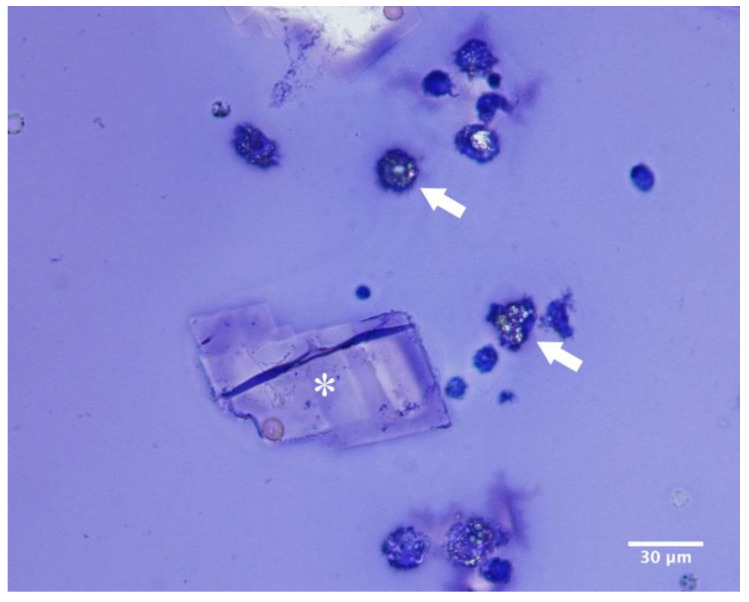
Case 2: Fluid from cyst of the third eyelid. In the background there is abundant bluish finely granular material (proteinaceous material) and some crystals (asterisk). Numerous foamy macrophages (arrows) are present (Diff-Quick stain, 40×).

**Figure 10:**
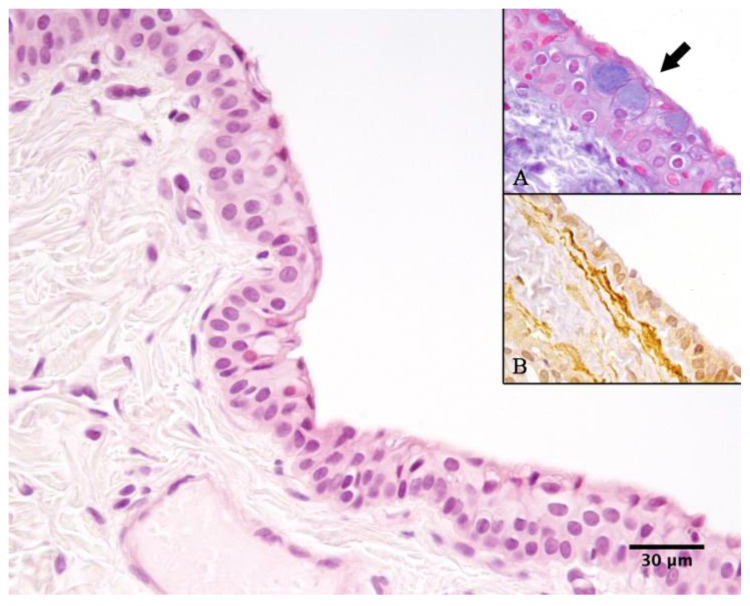
Case 2: Cyst of the third eyelid. The wall of the cyst is characterized by a non-ciliated cuboidal epithelium. (Hematoxylin and Eosin stain, 40×). Insert (**A**): Within the epithelium there are disseminated Alcian blue-positive goblet cells (arrow, Alcian blue stain, original magnification 40×). Insert (**B**) Subepithelial SMA-positive cells (in brown) are evident (Immunohistochemistry with HRP method for SMA, original magnification 40×).

Cytology of the cystic fluid and histopathology of the cyst wall showed similar findings to case no. 1 (Figure 9 and Figure 10).

## 4. Cytological, Histological, and Immunohistochemical Characterization of the Cysts

Cyst fluid from both cases 1 and 2 was submitted for cytological examination, revealing abundant proteinaceous material along with numerous macrophages, polygonal crystals, and occasional epithelial cells (Figure 9).

Histological examination, performed by an expert pathologist (FP), of the cysts from both cases revealed that the cyst walls were predominantly lined by a double layer of non-ciliated cuboidal epithelium, occasionally forming multilayered regions (Figure 10). The epithelium was supported by a dense fibrovascular stroma, which was multifocally infiltrated by a low number of neutrophils, macrophages, and occasional lymphocytes and plasma cells. Alcian blue staining highlighted rare, scattered goblet cells interspersed among the cuboidal epithelial cells (Figure 10A).

Immunohistochemistry for pan-cytokeratin (CK; AE1/AE3; Dako) and alpha-smooth muscle actin (SMA; 1A4; Dako, Carpinteria, CA, USA) was performed. Paraffin-embedded tissue sections (3 µm thick) were processed using the MACH1 Universal HRP Polymer Detection Kit (Biocare Medical LLC., Concord, CA, USA). Following deparaffinization, antigen retrieval was carried out using citrate buffer (pH 6) at 98 °C for 20 min. Endogenous peroxidase activity was blocked for 15 min at room temperature, followed by incubation with Background Sniper (Biocare Medical LLC.) for 30 min to minimize non-specific binding. Primary antibodies were diluted in phosphate-buffered saline (PBS, both 1:200) and incubated overnight at 4 °C. The MACH1 mouse probe was then applied for 20 min, followed by incubation with HRP polymer for 30 min, both at room temperature. Between each step, slides were washed in 0.01 M PBS (pH 7.2–7.4). Immunoreactivity was visualized using 3,3′-diaminobenzidine (DAB) as a chromogen, and sections were counterstained with Carazzi’s hematoxylin before coverslipping [25]. The cyst-lining epithelium showed strong cytoplasmic positivity for CK, while multiple layers of SMA-positive spindle cells were observed multifocally directly beneath the epithelium (Figure 10B).

To further characterize the SMA-positive spindle cells located directly beneath the cyst-lining epithelium, double-color immunofluorescence for SMA and p63 (a myoepithelial cell marker) was performed on the sample from case 2. After deparaffinization, antigen retrieval was performed by incubating the tissue sections in citrate buffer (pH 6) at 98 °C for 20 min. Sections were then incubated overnight at 4 °C in a humidified chamber with a primary antibody against SMA (1:200 dilution; clone 1A4; Dako). The following day, a FITC-conjugated rabbit anti-mouse secondary antibody (1:50 dilution; Jackson ImmunoResearch, Bar Harbor, ME, USA) was applied for 2 h at room temperature. Subsequently, a second primary antibody targeting p63 (1:50 dilution; clone 4A4; Abcam, Cambridge, UK) was applied and incubated overnight at 4 °C. A TRITC-conjugated rabbit anti-mouse secondary antibody (1:50 dilution; Jackson ImmunoResearch) was then applied for 2 h at room temperature. After each incubation step, slides were rinsed thoroughly with phosphate-buffered saline (PBS). Finally, sections were mounted using a 1:1 mixture of glycerol and PBS.

**Figure 11:**
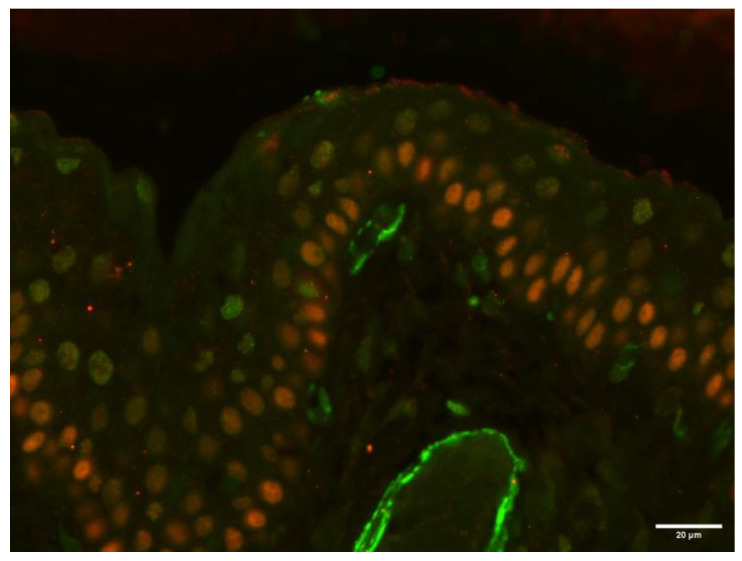
Case 2: Cyst of the third eyelid. The basal epithelial cells lining the cyst show weak p63 immunoreactivity (red), while the underlying SMA-positive (green) spindle cells are negative for p63 (double-color immunofluorescence for SMA and p63; original magnification, 40×).

The analysis revealed that the SMA-positive cells lacked p63 expression, indicating a non-myoepithelial phenotype (Figure 11).

## 5. Discussion

Pathogenetic theories of dacryops include a dysfunction of the rich nerve plexus around the ductules, periductular inflammation that induces scarring, a failure of ductular “neuromuscular” contractility, and IgA hypersecretion with an osmotic effect [23,24]. However, in all species, the exact pathogenesis of dacryops remains obscure.

The main contributing factor to the lack of clear knowledge regarding the pathogenesis of dacryops is probably the scarce information about the cell linings of the lacrimal cysts. It has been well established that a lacrimal cyst’ lining is composed of a double layer of cells, but its controversial if the outer epithelial layer of cells is a myoepithelium: finding myoepithelial cells could support the pathogenetic theory of failure of ductular “neuromuscular” contractility [23].

In a retrospective study in human medicine, immunohistochemical investigations were conducted in 15 examples of dacryops of 14 patients, compared to the normal lacrimal gland [23]. Smooth muscle actin (SMA)-positive myoepithelial cells were found in the acini of the normal glands but not in the normal ducts or dacryops epithelium [23]. Authors sometimes described in human dacryops a thin smooth muscle wall that was SMA-positive, but it was found in the fibrous wall of the dacryops always separated from the nonreacting epithelial lining by a mantle of collagen, and they speculated that it was due to neighboring vascular channels with SMA-positive myofibroblasts [23]. Since normal ducts and dacryops showed no immunohistochemical evidence for the presence of myoepithelial cells, the authors concluded that pathogenetic theories of dacryops that implicate a failure of ductular ‘‘neuromuscular’’ contractility must be revised [23].

In dogs, the presence of myoepithelial cells in canine dacryops is controversial. Some authors reported in canine dacryops localized in the rostroventral aspect of the eyes in three dogs the presence of several layers of SMA-positive “attenuated slender” cells directly beneath the epithelium, assuming that these cells were myoepithelial [7]. Other authors also reported a dog with dacryops localized in the rostroventral aspect of the eye and SMA-positive cells in the outer layer near the ductal epithelium, hypothesizing the presence of myoepithelial cells, which they presumed to be consistent with the glandular duct [11]. However, in both reported cases, it may be speculated that such cells may be myofibroblasts or smooth muscle cells of vessels present directly beneath the epithelium that delineates the cystic lesion.

In our cases here described, the histological findings are consistent with previous reports of canine dacryops [7,8,9,10].

In addition, as described in previously reported cases of canine dacryops localized in the rostroventral aspect of the eyes [7,11] and in one horse with dacryops localized in a similar position, close to the nasolacrimal duct [17], we observed different layers of SMA-positive spindle cells directly beneath the epithelium lining the cyst. To confirm or exclude the presence of myoepithelial cells, we performed a double-color immunofluorescence in the epithelium of case 2 of canine dacryops presented here. In addition to the anti-SMA antibody, we used the anti-p63 antibody to selectively label myoepithelial cells. The analysis revealed that the SMA-positive cells lacked p63 expression, indicating a non-myoepithelial phenotype.

Our results highlight the absence of myoepithelial cells in case 2, supporting the hypothesis that the SMA-positive cells described directly beneath the epithelium of canine dacryops are more likely to be neighboring myofibroblasts or vascular smooth muscle cells.

To our knowledge, the expression of p63 by SMA-positive cells beneath the epithelial layer of dacryops has never been evaluated in veterinary literature before our report.

Our findings support the hypothesis that, also in dogs, the pathogenesis of dacryops should exclude failure of ductular “neuromuscular” contractility, as demonstrated in humans by Jacobiec [23].

Furthermore, in dogs it has been reported that the normal lacrimal ducts (but not the stratified squamous epithelium that lines the lumen of the normal canaliculus) do manifest a myoepithelium [7], in contrast with reports in human beings, in which smooth muscle actin (SMA)-positive myoepithelial cells were found in the acini of the normal glands but not in the normal ducts [23]. Further studies are needed to clarify this anatomical feature.

In the cases here described, two adult dogs, we can theorise that the etiology of the lacrimal cysts reported was presumably either traumatic or inflammatory in origin.

Most histological studies of dacryops (ours included) in all species describe mild inflammation of the cyst wall, strengthening the hypothesis that periductal inflammation may be responsible for hypersecretion [24]. Furthermore, some authors hypothesized that chronic inflammation, leading to an immune response, may stimulate the adjacent plasma cells to produce IgA into the lumen, resulting in cyst formation by an osmotic effect [24]. Finally, in humans it was suggested that a congenital anomaly or intrinsic imbalance in the secretory composition may lead to cyst formation in predisposed individuals [24]: This theory could explain the etiology of dacryops found in young dogs, with a higher prevalence in certain breeds [11].

Interestingly, case 1 we report represents the first case of canine dacryops associated with unilateral entropion, although in humans these cysts lead to eyelid conformational abnormalities such as entropion [19]. It is possible to hypothesize that the inversion of the lid margin toward the globe in the affected eye was due to a decrease in orbital support of the eyelid and enophthalmos after chronic irritation from the dacryops. It was reported in an 8-month-old Neapolitan Mastiff dog to have a congenital dacryops of the lacrimal gland associated with hyperplasia and prolapse of the superficial gland of the nictitating membrane and bilateral macropalpebral fissure with ‘diamond eye’ conformation [9].

In case 1, the dacryops was closely related to the nasolacrimal system, and computed tomography dacryocystography was helpful to confirm the diagnosis and plan surgery.

The lacrimal system has been previously evaluated with computed tomographic dacryocystography in dogs and cats [26,27,28,29]. In our case, this diagnostic examination allowed us to locate a normal nasolacrimal structure without any communication with the cystic lesion that was closely adherent to the maxillary bone. Pressure due to the secretion of fluid can be the suspected pathophysiology that causes expansion of the cyst and erosion of the adjacent bone.

Surgical removal of these lesions is usually curative [7,8,9,17,30].

Canaliculops may, however, be treated with marsupialization [21], and cystorhinostomy (nasal marsupialization) was performed in two cases of ventromedial orbital dacryops in dogs [10]. Similarly, marsupialization was performed in three dogs affected by cysts developed after surgical repositioning of the nictitating membrane gland protrusion using the conjunctival pocket technique [31]. Finally, intralesional 1% polidocanol therapy was successful in resolving a suspected nasolacrimal duct cyst that extended into the caudal nasal cavity adjacent to the orbit in a 5-year-old castrated male Golden Retriever [28]. However, in this study, bioptic samples of the cystic structure were non-diagnostic, and the tissue of origin of the cyst could not be confirmed histologically or immunohistochemically [28]. The same treatment of intralesional 1% polidocanol injection was applied in three dogs affected by ventromedial orbital dacryops, but it failed in two of them [10].

## 6. Conclusions

Lacrimal cysts have to be considered in the differential diagnosis of periorbital masses in dogs. Surgical removal of these conditions is curative, and no recurrence has been reported. When the dacryops is closely related to the nasolacrimal system, a computed tomographic dacryocystogram is the gold standard for establishing the diagnosis and planning surgery.

Finally, our immunohistochemical findings support the conclusion that in dogs ’the pathogenesis of dacryops should exclude failure of ductular “neuromuscular” contractility.

## Data Availability

No new data were created or analyzed in this study. Data sharing is not applicable to this article.
